# *HTRA1* promoter variant differentiates polypoidal choroidal vasculopathy from exudative age-related macular degeneration

**DOI:** 10.1038/srep28639

**Published:** 2016-06-24

**Authors:** Tsz Kin Ng, Xiao Ying Liang, Timothy Y. Y. Lai, Li Ma, Pancy O. S. Tam, Jian Xiong Wang, Li Jia Chen, Haoyu Chen, Chi Pui Pang

**Affiliations:** 1 Department of Ophthalmology and Visual Sciences, The Chinese University of Hong Kong, Hong Kong; 2 The Joint Shantou International Eye Center of Shantou University and The Chinese University of Hong Kong, Shantou, China

## Abstract

Exudative age-related macular degeneration (AMD) and polypoidal choroidal vasculopathy (PCV) share similar abnormal choroidal vasculature, but responses to treatments are different. In this study, we sequenced the whole *HTRA1* gene and its promoter by direct sequencing in a Hong Kong Chinese PCV cohort. We identified rs11200638, c.34delCinsTCCT, c.59C>T, rs1049331 and rs2293870 significantly associated with PCV. Notably, rs2672598 was significantly associated with exudative AMD (*p* = 1.31 × 10^−4^) than PCV (*p* = 0.11). Logistic regression indicated that rs2672598 (*p* = 2.27 × 10^−3^) remained significant after adjusting for rs11200638 in exudative AMD. Moreover, the rs11200638-rs2672598 joint genotype AA-CC conferred higher risk to exudative AMD (43.11 folds) than PCV (3.68 folds). Promoter analysis showed that rs2672598 C-allele showed higher luciferase expression than wildtype T-allele (*p* = 0.026), independent of rs11200638 genotype (*p* = 0.621). Coherently, vitreous humor HTRA1 expression with rs2672598 CC genotype was significantly higher than that with TT genotype by 2.56 folds (*p* = 0.02). Furthermore, rs2672598 C-allele was predicted to alter the transcription factor binding sites, but not rs11200638 A-allele. Our results revealed that *HTRA1* rs2672598 is more significantly associated with exudative AMD than PCV in *ARMS2/HTRA1* region, and it is responsible for elevated *HTRA1* transcriptional activity and HTRA1 protein expression.

Age-related macular degeneration (AMD) is a progressive macular disease and a major cause of irreversible blindness in the elderly population^1^. At the advanced stage, it can be classified into exudative and non-exudative AMD^2^. Exudative AMD, characterized by choroidal neovascularization (CNV), shares similar phenotype with polypoidal choroidal vasculopathy (PCV). Both of them involve abnormal vasculopathies arising from the choroidal vasculature leading to sub-retinal serous exudation and hemorrhage^3^,^4^. PCV is characterized by the polypoidal structures beneath the relatively intact Bruch’s membrane, branching from abnormal inner choroidal vasculature^5^. The polypoidal structures can be visualized by indocyanine green angiography (ICGA), a diagnostic tool for differentiating PCV lesion from CNV. PCV and CNV are different in their risk factors^6^, clinical manifestations^7^, natural courses^5^, responses to treatment^8^, and the overall visual prognosis^9^. PCV occurs predominately in Asian populations and tends to be younger^6^. Its disease course is relatively benign with less drusen but a tendency of relapse^5^. Without treatment, the visual outcome of PCV is usually guarded^10^. Patients with AMD, however, tend to present with a more aggressive course and uncontrollable visual deterioration^6^. In addition, exudative AMD responds better to anti-vascular endothelial growth factor (VEGF) treatment, whereas PCV better to photodynamic therapy (PDT)^9^,^11^,^12^.

Some reports propose that PCV could be a variant of typical CNV and regarded as one of the subtypes of exudative AMD^6^,^13^. This could be reflected by their similar genetic and environmental factors. Multiple genetic variants have exhibited significant association with both exudative AMD and PCV^6^. These include the complement factor H (*CFH*) gene, age-related maculopathy susceptibility 2 (*ARMS2*)/high temperature requirement factor A1 (*HTRA1*) locus and complement component 2 (*C2*)/factor B (*CFB*) genes^14^. Previously, we screened the entire *ARMS2* gene by direct sequencing in our Hong Kong Chinese exudative AMD and PCV cohort^15^. Although significant differences in the genotypic distributions of 11 polymorphisms in *ARMS2* gene were found between exudative AMD and PCV, all of the associated polymorphisms showed similar trends. Since the *ARMS2* and *HTRA1* genes are in complete linkage disequilibrium (LD), it is also important to delineate the association pattern of *HTRA1* in PCV patients. Three *HTRA1* haplotypes are associated individually with typical AMD and PCV in a Japanese study although no difference between them was reported^16^. Therefore, the differential association between exudative AMD and PCV remains equivocal. The association of other *HTRA1* variants in PCV should be investigated. In this study, 9 exons, intron-exon junctions and the promoter region of *HTRA1* gene were sequenced in our Hong Kong Chinese PCV cohort with a view to clarify the association of *HTRA1* with PCV.

## Results

### Sequence variant association analysis

**Figure 1:**
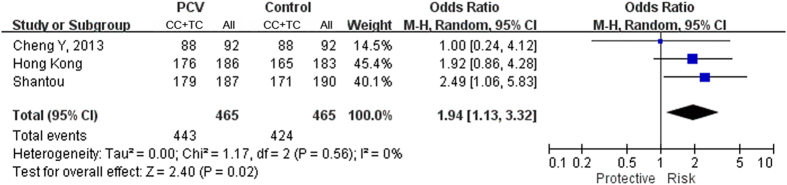
Meta-analysis of rs2672598 in 3 Chinese PCV cohorts. Three Chinese cohorts (Hong Kong, Shantou and Beijing) were included in the meta-analysis under dominant model. Random effect was chosen. Squares indicate study-specific OR. The size of the box is proportional to the weight of the study. Horizontal lines indicate 95% CI. Diamond indicates summary OR with its corresponding 95% CI.

Totally 26 polymorphisms in *HTRA1* were identified in PCV patients (Table 1). Four of them, IVS5+76_79delGTTT, rs79778361 (IVS6+111G>A), rs76357476 (IVS7+149C>G) and rs2293871 (IVS8-36C>T), were excluded from further analysis since they did not follow the Hardy-Weinberg equilibrium. Among the remaining 22 polymorphisms, 10 uncommon variants (minor allele frequency <3%) and 6 common variants did not showed significant associations. They were excluded in subsequent analysis. The remaining 6 variants were further analyzed for their corresponding ORs under dominant model (Table 2). Two SNPs, c.34delCinsTCCT and rs2672598 (−487T>C), were also included in the analysis since they showed significant association with exudative AMD. Four variants were associated with exudative AMD: rs11200638 (−625G>A; *p* = 1.16 × 10^−5^, OR = 3.21, 95% confident intervals (CI): 1.88–5.50), c.34delCinsTCCT (L12insS; *p* = 0.001, OR = 0.13, 95% CI: 0.13–0.57), rs1049331 (c.102C>T, A34A; *p* = 1.16 × 10^−5^, OR = 3.21, 95% CI: 1.88–5.50), and rs2293870 (c.108G>T, G36G; *p* = 1.16 × 10^−5^, OR = 3.21, 95% CI: 1.88–5.50) as well as PCV (rs11200638: *p* = 0.005, OR = 2.01, 95% CI: 1.23–3.28; c.34delCinsTCCT: *p* = 0.029, OR = 0.34, 95% CI: 0.13–0.90; rs1049331: *p* = 0.009, OR = 1.91, 95% CI: 1.17–3.11; and rs2293870: *p* = 0.009, OR = 1.91, 95% CI: 1.17–3.11). None of these variants showed significant differences between AMD and PCV under dominant model (*p* > 0.05). Instead, a significant differential association (*p* = 0.015, OR = 5.48, 95% CI: 1.19–25.37) was detected in rs2672598. It was strongly associated with exudative AMD (*p* = 1.31 × 10^−4^, OR = 10.53, 95% CI: 2.41–46.04) but not associated with PCV (*p* = 0.106). Our result is coherent to a recent report in northern China that rs2672598 is associated with exudative AMD (*p* = 4.60 × 10^−3^), but not PCV (*p* = 0.24)^17^. In order to further illustrate the association of rs2672598 with PCV, we genotyped another PCV cohort in southern China. Unlike the Hong Kong and Beijing cohort, rs2672598 was associated with PCV in Shantou cohort (*p* = 0.031, OR = 2.49, 95% CI: 1.06–5.83). The meta-analysis of the 3 Chinese cohorts at rs2672598 also showed a significant association with PCV (*p* = 0.02, OR = 1.94, 95% CI: 1.13–3.32; Fig. 1). In addition, c.59C>T (A20V) was significantly associated with PCV (*p* = 0.004, OR = 0.49, 95% CI: 0.30–0.79), but not with exudative AMD (*p* = 0.13).

### Haplotype analysis

**Figure 2:**
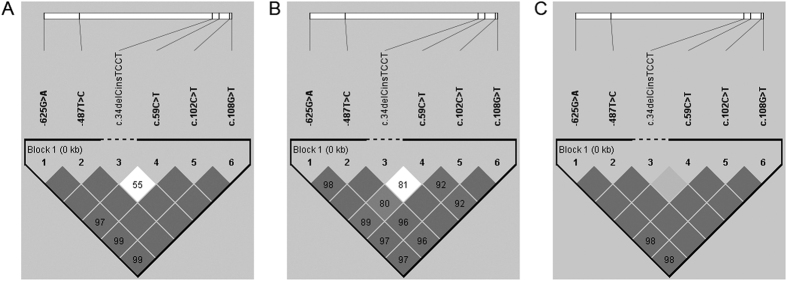
Haplotype block structure for the associated *HTRA1* polymorphisms. The haplotype analysis revealed linkage disequilibrium (LD) blocks lying across all of the associated *HTRA1* polymorphisms except for the c.34delCinsTCCT in **(A)** exudative AMD, in **(B)** PCV and in **(C)** control subjects. The displayed value of the LD was D’ (the value of D’ × 100, threshold = 0.8), and the r^2^ threshold was 0.8.

LD and haplotype-based analyses of the 6 associated variants showed that rs11200638, rs1049331, and rs2293870 were in perfect LD (r^2^ > 0.95) in both exudative AMD and PCV. LD blocks across all variants were observed in exudative AMD (Fig. 2A), PCV (Fig. 2B) and in control subjects (Fig. 2C), except c.34delCinsTCCT probably because of its low frequency. It was excluded for haplotype-based association analysis. Three associated haplotypes defined by these 5 variants were predicted (Table 3). Haplotype ACCTT, defined by the risk alleles, was associated with increasing risk in both exudative AMD (*p* = 8.70 × 10^−14^, OR = 3.10, 95% CI: 2.29–4.19) and PCV (*p* = 1.60 × 10^−4^, OR = 1.66, 95% CI: 1.24–2.22). Haplotype GTCCG, defined by the wildtype alleles, was associated with decreasing risk in exudative AMD (*p* = 5.35 × 10^−12^, OR = 0.25, 95% CI: 0.16–0.38), but not in PCV (*p* = 0.17). These 2 haplotypes were different between AMD and PCV (*p* < 0.001). Another haplotype, GCTCG, was associated with PCV (*p* = 0.04, OR = 0.57, 95% CI: 0.37–0.89), but not with exudative AMD (*p* = 0.363).

### Joint genotype analysis

**Figure 3:**
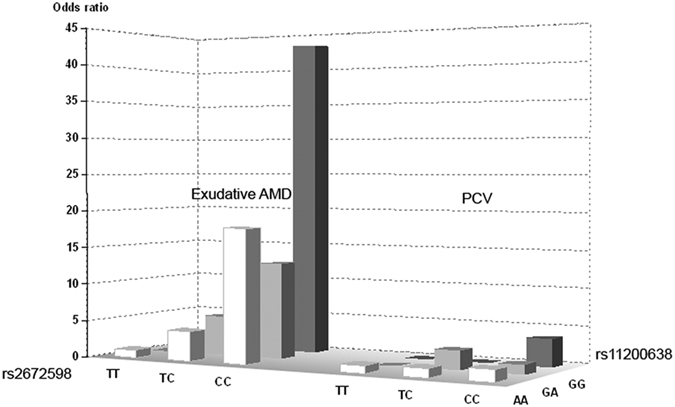
The odds ratio plot of rs11200638–rs2672598 joint genotypes. The rs11200638-A and rs2672598-C were the risk alleles. The odds ratio plot revealed the highest joint odds ratio of 43.11 from the AA-CC joint genotype in exudative AMD group. In addition, >10-fold higher risk was observed in exudative AMD, comparing to PCV.

The 4 associated variants between exudative AMD and PCV were examined by joint genotype analysis SNP rs11200638 was used as a proxy because it was in perfect LD with other two SNPs (rs1049331 and rs2293870). Therefore, only rs11200638 and rs2672598 were included in the analysis. The results of the joint genotypes included homozygous carriers of rs2672598-C risk allele. All genotypes of rs11200638 were associated with exudative AMD (*p*_AA-CC_ = 4.31 × 10^−8^, *p*_GA-CC_ = 3.00 × 10^−3^ and *p*_GG-CC_ = 2.00 × 10^−3^, respectively). Significant differences were found between exudative AMD and PCV (*p*_AA-CC_ = 4.00 × 10^−3^, OR = 11.70, 95% CI: 1.45–94.53; *p*_GA-CC_ = 0.01, OR = 10.16, 95% CI: 1.22–84.81 and *p*_GG-CC_ = 0.02, OR = 11.25, 95% CI: 1.17–108.41, respectively). However, only the homozygous risk allele carriers were associated with PCV (*p* = 3.00 × 10^−3^). The highest OR in homozygous risk allele joint genotypes for exudative AMD (OR = 43.11, 95% CI: 5.56–334.49) has 11.7-fold higher risk (*p* = 4.00 × 10^−3^) than for PCV (OR = 3.68, 95% CI: 1.51–8.99) (Fig. 3 and Supplementary Table 1). Logistic regression analysis showed that rs11200638 (*p* = 1.48 × 10^−4^) and rs2672598 (*p* = 2.27 × 10^−3^) were independently associated with exudative AMD after adjusting for age and gender. On the contrary, in PCV, rs11200638 remained significant (*p* = 9.05 × 10^−3^) but not rs2672598 (*p* = 0.20).

### HTRA1 promoter analysis

**Figure 4:**
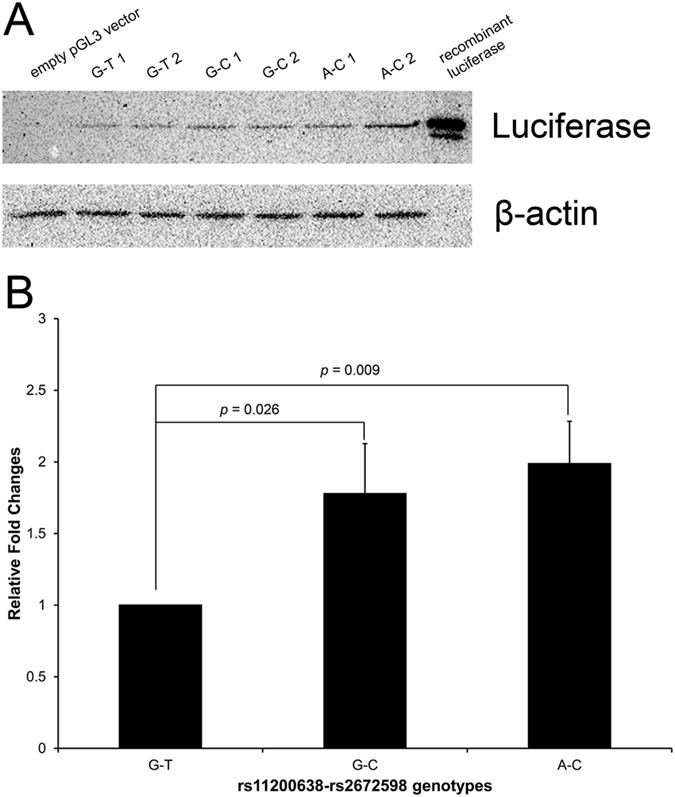
Luciferase expression driven by the *HTRA1* promoter with different *HTRA1* rs11200638-rs2672598 haplotypes. A 726-bp genomic fragment (from −658 to +68) containing the *HTRA1* promoter was cloned into empty pGL3-Basic vector. Human retinal pigment epithelial cell line ARPE-19 were transfected with different luciferase constructs for 72 hours. (**A**) Detection of luciferase expression was performed by immunoblotting. (**B**) Normalized luciferase intensities were calculated by dividing the quantified luciferase intensities by the housekeeping β-actin intensities. Two different clones of each haplotype were used in the transfection experiment. The wildtype rs11200638-rs2672598 haplotype (G-T) was set as a reference. Recombinant firefly luciferase was used as a positive control, whereas empty pGL3 was used as negative control.

Cloning the *HTRA1* promoter identified three rs11200638-rs2672598 haplotypes: G-T (wildtype), G-C and A-C. No A-T haplotype was found. Luciferase expression analysis showed that the rs11200638-rs2672598 haplotypes affect differential transcriptional activities of the *HTRA1* promoter (Fig. 4). Comparing to G-T haplotype, elevated transcriptional activity was observed in G-C and A-C haplotypes (*p* = 0.009, one-way ANOVA; Post-hoc tests adjusted by Tukey HSD: G-C vs G-T, *p* = 0.026, and A-C vs G-T, *p* = 0.009) with 1.78 ± 0.35 and 1.99 ± 0.30 fold increase, respectively. However, there was no difference in luciferase expression level between G-C and A-C haplotypes (*p* = 0.621), suggesting that A-allele of rs11200638 might not alter the transcriptional activity of the *HTRA1* promoter. Therefore, rs2672598 should be the variant that contributed to the altered transcriptional activity of the *HTRA1* promoter.

**Figure 5:**
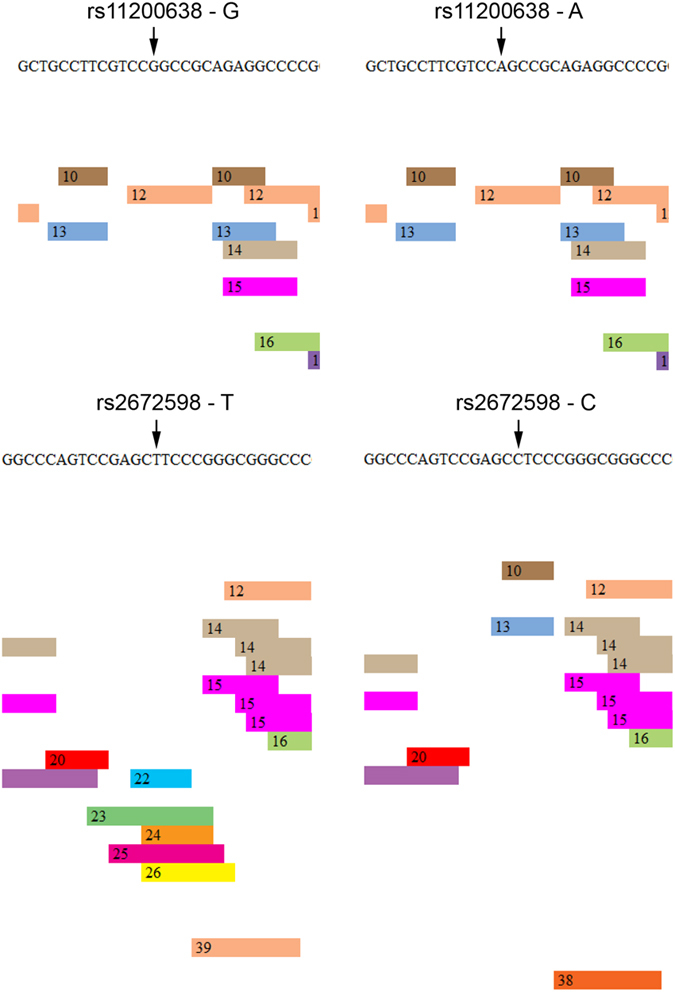
Transcription factor binding site prediction at rs11200638 and rs2672598 in the *HTRA1* promoter. DNA sequence of the cloned *HTRA1* promoter was used to predict the transcription factor binding sites. PROMO was used to predict the transcription factors that would bind to the region of rs11200638 and rs2672598 in the *HTRA1* promoter. Predictions with different alleles of rs11200638 and rs2672598 were performed. Transcription factors: 10: GR-alpha; 12: E2F-1; 13: AP-2αA; 22: STAT4; 23: NFκB; 24: c-Ets-1; 25: RelA; 26: Elk-1; 38: Sp1; 39: WT1.

Our luciferase-reporter analysis showed that transcription activity increased with rs2672598 C-allele but not rs11200638 A-allele. This phenomenon may be due to influences of transcription factor binding sites within these regions. With G-T haplotype as a reference, one transcription factor E2F-1 binding site was predicted at rs11200638, and one each of STAT4, NFκB, c-Ets-1, RelA, Elk-1 and WT1 binding sites were predicted at rs2672598 site (Fig. 5). When rs11200638 G-allele was replaced by A-allele, the transcription factor E2F-1 binding site was unchanged. In contrast, when rs2672598 T-allele was replaced by C-allele, all the transcription factor binding sites were abolished and changed to GR-alpha, AP-2αA and Sp1 binding sites. Bioinformatic analysis confirmed that rs2672598 contributed to the altered transcriptional activity of the *HTRA1* promoter.

### Correlation of rs2672598 genotype and HTRA1 expression in vitreous humor

**Figure 6:**
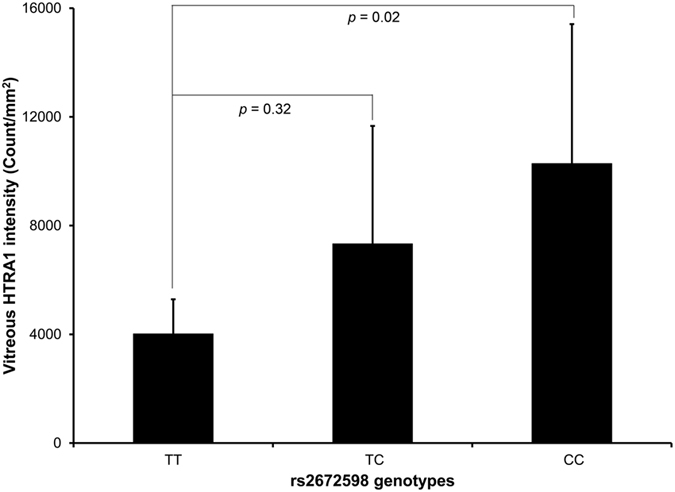
Correlation analysis of rs2672598 genotypes and HTRA1 protein expression in human vitreous humor samples. HTRA1 expressions in 55 vitreous humor samples were determined by immunoblotting, and expressed in intensity (count/mm^2^). Genotyping of rs2672598 polymorphism revealed TT genotype in 5 samples, TC genotype in 23 samples and CC genotypes in 27 samples. The HTRA1 expression of CC genotype (homozygous) was significantly higher than that of TT genotype (wildtype).

In order to validate the elevated transcriptional activity of rs2672598 C-allele, we performed the genotype-expression correlation analysis in vitreous humor collected from our previous study^18^, which HTRA1 protein expression has been determined. In this study, the rs2672598 genotype of the vitreous humor samples was determined, and 5 samples were in TT genotype, 23 samples in TC genotype and 27 samples in CC genotypes. The HTRA1 expression was higher in the vitreous humor with rs2672598 CC genotype than that with TT genotype by 2.56 folds (*p* = 0.009, one-way ANOVA; Post-hoc tests adjusted by Tukey HSD: CC vs TT, *p* = 0.02; Fig. 6). HTRA1 level of TC genotype is also higher than TT genotype by 2.03 folds but not statistically significant (*p* = 0.32). As we have previously shown than rs11200638 is not correlated with HTRA1 expression in vitreous humor^18^, this result confirmed that rs2672598 should be the variant leading to the differential *HTRA1* expression in human ocular samples.

## Discussion

PCV has been regarded as a different disease entity from AMD, with a distinct set of demographic, pathological and clinical characteristics^3^. However, emerging evidences suggest that PCV could be a variant of typical CNV^5^, sharing common genetic determinants and environmental risk factors^6^. Moreover, elevated serum C reactive protein and VEGF levels in CNV were also found in PCV^19^,^20^. In clinical practice, PCV usually responds better to PDT^8^,^11^, whereas exudative AMD responds better to anti-VEGF treatment^12^,^21^. Meanwhile, they share some common genetic determinants. *ARMS2* rs10490924 and *HTRA1* rs11200638 were associated with both exudative AMD and PCV in a Japanese study, in which neither allelic nor genotypic frequencies showed significant difference^22^. Similar association of rs10490924 with advanced AMD and PCV has also been reported in Caucasians^23^. In contrast, our previous study identified significantly different association of *ARMS2* polymorphisms between exudative AMD and PCV in the Chinese population^15^.

Results in this study revealed a *HTRA1* promoter variant (rs2672598) that is significantly associated with exudative AMD, but not PCV (Tables 1 and 2). Our result is coherent to a recent report in a northern Chinese population^17^. Although meta-analysis of the 3 Chinese cohorts at rs2672598 also showed a significant association with PCV (*p* = 0.02, OR = 1.94, 95% CI: 1.13–3.32; Fig. 1), rs2672598 is more significantly associated with exudative AMD than PCV as shown in Hong Kong (exudative AMD: *p* = 1.31 × 10^−4^; PCV: *p* = 0.11) and Beijing cohorts (exudative AMD: *p* = 4.60 × 10^−3^; PCV: *p* = 0.24). Furthermore, the joint genotype constructed by homozygous carriers of risk allele (rs2672598-C and rs11200638-A) were prone to exudative AMD (OR = 43.11, 95% CI: 5.56–334.49), conferring 11.7-fold higher risk (*p* = 4.00 × 10^−3^) when compared to PCV (OR = 3.68, 95% CI: 1.51–8.99; Fig. 3 and Supplementary Table 1). Moreover, joint genotypes constructed by rs2672598-CC and rs11200638-GA/GG conferred increasing AMD risk (OR = 12.86, 95% CI: 1.64–100.86; OR = 18.00, 95% CI: 2.00–161.83, respectively), but not associated with PCV. Logistic regression results further indicated differential roles of rs2672598 in exudative AMD and PCV. This SNP might contribute independently of rs11200638 to exudative AMD but not PCV.

*HTRA1* has its own role in both AMD and PCV^24^,^25^, governed by different molecular determinants. In this study, rs2672598, located in the promoter region of *HTRA1*, not only showed differential associations between AMD and PCV, but also altered the transcriptional activity of the *HTRA1* promoter. Higher transcriptional activity of the *HTRA1* promoter was observed in the constructs with rs2672598 C-allele rather than that with rs11200638 A-allele (Fig. 4), suggesting that the rs2672598 C-allele, not rs11200638 A-allele, is responsible for the alteration in the transcriptional activity of the *HTRA1* promoter. Our luciferase-reporter analysis agreed with the transcription factor binding site prediction analysis (Fig. 5). No alteration of transcription factor binding site was found in rs11200638 A-allele when compared to the wildtype G-allele. In contrast, the transcription factor binding sites in rs2672598 C-allele were totally different from that in the wildtype T-allele. This further confirmed that rs2672598 should be the variant contributed to the altered transcriptional activity of the *HTRA1* promoter. In order to validate the results of *in vitro* transcriptional activity, we performed a correlation analysis on the HTRA1 protein expression in vitreous humor with the rs2672598 genotypes. Our results showed that HTRA1 expression was higher in the vitreous humor with rs2672598 CC genotype than that with TT genotype by 2.56 folds (*p* = 0.02; Fig. 6). As we previously showed than rs11200638 is not correlated with HTRA1 expression in vitreous humor^18^, the vitreous humor expression results in this study not only validate our findings in the promoter analysis, but also confirm that rs2672598 is responsible for the higher HTRA1 expression in human ocular samples. Our luciferase activity and vitreous humor expression results of rs11200638 are similar to previous reports that there is no significant change of *HTRA1* gene expression level in human retina among genotypes of *HTRA1* rs11200638, rs1049331 and rs2293870, and *ARMS2* rs10490924 and the del443ins54 variant^26^,^27^.

In summary, our results revealed that a *HTRA1* promoter variant, rs2672598, is more significantly associated with exudative AMD than PCV. It should be responsible for elevated transcriptional activity in the *HTRA1* promoter.

## Methods

### Study subjects

We recruited 188 PCV patients from the Prince of Wales Hospital Eye Centre and the Hong Kong Eye Hospital in Hong Kong. Together with 195 exudative AMD patients and 183 normal controls reported in our previous study^28^, a total of 534 unrelated Han Chinese were included (Supplementary Table 2). For validation, we recruited 187 PCV patients and 190 control subjects from the Joint Shantou International Eye Center of Shantou University and The Chinese University of Hong Kong, Shantou, China. All study subjects, including patients and controls, were given comprehensive ophthalmic evaluations and examined by clinical ophthalmologists. Both fluorescein angiography (FA) and ICGA were performed in the exudative AMD and PCV patients. AMD was graded according to an international classification and grading system^29^. Patients with exudative AMD had non-drusenoid RPE detachment, choroidal neovascularization, serous or hemorrhagic retinal detachments, sub-retinal or sub-RPE hemorrhage or fibrosis. PCV patients had sub-retinal red or orange nodules and hemorrhagic pigment epithelial detachment and characteristic sacculated vascular abnormalities in the inner choroid as visualized on ICGA. The diagnosis of PCV was distinguished from AMD by FA and ICGA staining^30^. PCV patients with geographic atrophy or early signs of AMD were excluded, together with patients who were difficult to be clearly differentiated (at late stage of disease that possess fibrosis and disciform scar or at advanced stage that present with intensive hemorrhage). The control subjects were recruited from elderly people greater than 60 years. They did not have any identifiable signs of AMD, PCV or other major eye diseases, except for mild senile cataract and slight refractive errors ranging from −1.5 to +1.5 diopters. The study protocol, approved by the Ethics Committee for Human Research at the Chinese University of Hong Kong, is in accordance with the tenets of the Declaration of Helsinki. Informed consent was obtained from each study subject.

### Sequence analysis

Genomic DNA from whole blood was extracted (Qiagen QIAamp DNA Blood Mini kit, Qiagen, Hiden, Germany) according to the supplier’s instructions. The 9 exons, intron-exon junctions of *HTRA1* (ENSG00000166033) and its promoter region were screened by polymerase chain reaction (PCR) with specific primers followed by direct sequencing (BigDye Terminator Cycle Sequencing Reaction Kit, v3.1; Applied Biosystems, Foster City, CA) on a DNA sequencer (ABI 3130XL, Applied Biosystems)^28^.

### Association analysis

All the identified polymorphisms were assessed for Hardy-Weinberg equilibrium using χ^2^ analysis. Allelic and genotypic distributions for association between PCV and controls, between exudative AMD and controls, and between exudative AMD and PCV) were compared using the χ^2^ test or Fisher’s exact test (SPSS, version 16.0; SPSS Science, Chicago, IL). Logistic regression was used to examine the association profiles of the associated polymorphisms in *HTRA1* between exudative AMD and PCV (SPSS). LD and haplotype-based association analyses were performed based on Haploview, version 4.2; http://www.broadinstitute.org/)^31^. Multiple testing correction was performed by permutation test (n = 10,000).

For the meta-analysis, the Review Manager software (RevMan, version 5.2, The Cochrane Collaboration, Copenhagen, Denmark) was used to generate the *Z* scores. The exact *P* values with the *Z* scores was then calculated using R (v3.0.0, http://cran.r-project.org/). The *I2* statistic was used to assess the heterogeneity among studies, which corresponds to no (<25%), low (25–50%), moderate (50–75%), and high heterogeneity (≥75%). We adopted the random effects model in the meta-analysis. Summary *p* < 0.05 was considered statistically significant.

### Joint genotype analysis

The genotype combinations of rs11200638 and rs2672598 in exudative AMD, PCV and control subjects were counted. Their corresponding odds ratios (OR) were calculated by the χ^2^ test (SPSS). The ORs were compared to the baseline genotype of the two genes that showed the lowest frequency of the disease risk alleles (homozygous G carrier of rs11200638 with homozygous T carrier of rs2672598).

### Promoter analysis

A 726-bp genomic fragment (from −658 to +68) containing the *HTRA1* promoter was cloned into empty pGL3-Basic vector, pGL3 (Promega, Madison, WI) between the *Nhe*I and *Bgl*II sites (OriGene Technologies, Rockville, MD). Genomic DNA of the study subjects were amplified by PCR (*Ex Taq* DNA Polymerase, TakaRa, Japan): Forward primer (5′-TAATGCTA GCTCTCTGCGAATACGGACACG), reverse primer (5′-TAATAGATCTGGGAGAGTGCAG GAGGG). Constructs with different rs11200638-rs2672598 haplotypes were generated. The cloned sequence of all constructs was verified by direct sequencing.

Human retinal pigment epithelial cell line ARPE-19 (American Type Culture Collection, Manassas, VA)^32^ was cultured in Dulbecco’s modified Eagle’s medium and F-12 nutrient mixture (DMEM/F-12) supplemented with 10% fetal bovine serum (Gibco BRL, Rockville, MD). Cells were plated in 60 mm tissue culture dishes at a density of 2–3 × 10^5^ cells per dish one day before transfection. After 24 hours, cells were transfected with 2.5 μg of luciferase constructs in 7.5 μl FuGene HD transfection reagent (Roche, Indianapolis, IN) per dish. Empty pGL3 was used as negative control. At 72 hours after transfection, cell lysates were extracted by RIPA reagent (Sigma-Aldrich, St. Louis, MO) for detection of luciferase expression.

Luciferase expression was detected by immunoblotting according to our published method^33^. Briefly, the denatured cell lysates of the transfected cells were resolved on 10% SDS-polyacrylamide gel and electro-transferred to nitrocellulose membranes for probing with a mouse monoclonal primary antibody against firefly luciferase (LifeSpan BioSciences, Seattle, WA) and a secondary antibody against mouse IgG conjugated with horseradish peroxidase (Santa Cruz Biotechnology, Dallas, TX). Signals were detected by the enhanced chemiluminescence (ECL) system (Amersham Pharmacia, Cleveland, OH) and quantified by ChemiDoc (BioRad, Hercules, CA). Normalized luciferase intensities were calculated by dividing the quantified luciferase intensities by the housekeeping β-actin intensities. Two different clones of each haplotype were used in the transfection experiment. Triplicated experiments were performed. The wildtype rs11200638-rs2672598 haplotype (G-T) was set as a reference. One-way Analysis of Variance (ANOVA) with post-hoc tests adjusted by Tukey HSD (SPSS) was used to compare the means among different groups. Recombinant firefly luciferase (Promega) was used as a positive control.

DNA sequence of the cloned *HTRA1* promoter was used to predict the transcription factor binding sites. We predicted the transcription factors that would bind to the region of rs11200638 and rs2672598 in the *HTRA1* promoter by PROMO (version 3.0.2; http://alggen.lsi.upc.es/cgi-bin/promo_v3/promo/promoinit.cgi?dirDB=TF_8.3)^34^. Predictions with different alleles of rs11200638 and rs2672598 were performed.

### Correlation analysis of rs2672598 genotype and HTRA1 expression in vitreous humor

Fifty-five unrelated Chinese patients underwent ocular surgeries at the Prince of Wales Hospital in Hong Kong and the Joint Shantou International Eye Center (JSIEC) of Shantou University and the Chinese University of Hong Kong were recruited and given complete ophthalmoscopic examinations^18^. In all patients, a standard three-port pars plana vitrectomy was performed as a part of the regular surgical procedures. Undiluted vitreous humor samples (0.5–1 ml) were collected into sterile tubes at the time of surgery and aliquots were rapidly frozen at −80 °C until assay.

HTRA1 protein expression has been previously determined^18^. Briefly, total protein concentrations in the vitreous samples were measured by Protein assay (BioRad, Hercules, CA). Equal amount of total protein (10 ug) for each denatured vitreous humor samples were resolved on 12.5% SDS-polyacrylamide gel and electro-transferred to nitrocellulose membranes for probing with the mouse monoclonal antibody against HTRA1 (R&D Systems Inc., Minneapolis, MN), and secondary antibody against mouse IgG conjugated with horseradish peroxidase (Jackson Immuno. Res., West Grove, PA). The signals were detected by enhanced chemiluminescence (ECL) (Amersham Pharmacia, Cleveland, OH) and the band intensities quantified by Quantity One^®^ Image Analysis software (BioRad).

DNA was extracted from all of the vitreous humor samples using a commercially available DNA extraction kit (Qiagen, Germany). The *HTRA1* rs2672598 genotypes were determined in all 55 samples by polymerase chain reaction (PCR) and direct DNA sequencing as described above^28^.

## Additional Information

**How to cite this article**: Ng, T. K. *et al*. *HTRA1* promoter variant differentiates polypoidal choroidal vasculopathy from exudative age-related macular degeneration. *Sci. Rep.* **6**, 28639; doi: 10.1038/srep28639 (2016).

## Supplementary Material

Supplementary Information

## Figures and Tables

**Table 1:** The *HTRA1* polymorphisms identified in PCV subjects.

Polymorphism	db SNP ID	Codon change	Genotypic Frequencies										
			PCV (n = 188)			AMD (n = 195)			Control (n = 183)			*p*_G_	*p*_A_
−625G>A	rs11200638	–	70	84	33	109	63	23	38	90	55	1.00 × 10^−4^	7.49 × 10^−4^
−502C>T	Reported	–	0	15	173	0	14	181	0	20	163	0.33	0.34
−497C>T	Reported	–	0	7	181	0	6	189	0	8	175	0.75	0.75
−487T>C	rs2672598	–	109	67	10	162	31	2	97	68	18	0.23	0.13
c.34delCinsTCCT	Novel	L12insS	0	5	183	0	2	193	0	16	167	2.90 × 10^−2^	3.10 × 10^−2^
c.59C>T	Novel	A20V	7	30	148	3	42	150	2	56	125	3.70 × 10^−3^	0.01
c.102C>T	rs1049331	A34A	70	84	33	108	64	23	38	90	55	1.00 × 10^−4^	9.43 × 10^−4^
c.108G>T	rs2293870	G36G	70	84	33	108	64	23	38	90	55	1.00 × 10^−4^	9.43 × 10^−4^
c.IVS1-176C>G	rs12267142	–	0	17	170	0	19	176	0	25	158	0.17	0.18
c.569G>A	Novel	R190H	0	1	186	0	0	195	0	0	183	1.00	1.00
c.IVS2+100C>T	rs80158665	–	0	4	183	0	4	191	0	8	175	0.23	0.23
c.IVS2+216A>G	Reported	–	0	1	186	0	0	195	0	1	182	1.00	1.00
c.IVS2+317C>T	Reported	–	0	2	185	0	2	193	0	1	182	1.00	1.00
c.672C>T	Novel	N224N	0	1	186	0	0	195	0	0	183	1.00	1.00
IVS3+93C>T	rs2239586	–	30	73	84	19	89	87	22	92	69	0.09	0.65
IVS3+167G>A	rs2239587	–	30	73	84	19	89	87	22	92	69	0.09	0.65
IVS4+99C>T	rs2672582	–	38	76	73	35	105	55	30	86	67	0.41	0.85
IVS5+76_79delGTTT	Reported	–	7	56	124	0	69	126	0	48	135	–	–
IVS5+169G>A	rs2672583	–	39	77	71	38	105	52	28	87	68	0.30	0.51
IVS6+58C>T	Novel		0	1	186	0	0	195	0	0	183	1.00	.001
IVS6+111G>A	rs79778361	–	2	42	144	5	39	151	1	57	125	–	–
IVS6+115C>G	rs2672585	–	39	77	72	33	107	55	28	88	67	0.27	0.60
IVS7+149C>G	rs76357476	–	2	43	143	5	40	150	1	57	125	–	–
c.1221C>T	rs11538140	D407D	0	3	184	0	3	192	0	5	178	0.46	0.50
IVS8+14G>A	rs2272599	–	71	76	40	53	107	35	67	85	31	0.42	0.67
IVS8−36C>T	rs2293871	–	34	81	73	28	90	77	24	102	57	–	–

pG = genotypic p-value for PCV; pA = allelic p-value for PCV. The genotypic distribution was presented as homozygous/heterozygous/wild type. The rare variants in PCV (R190H) and exudative AMD (R59P, V417I) did not showed significant association either in single marker or grouped analysis.

**Table 2:** The odds ratio analysis of associated *HTRA1* polymorphisms.

Polymorphism	db SNP ID	Genotypic frequency			AMD		PCV		AMD-PCV	
		AMD (n = 195)	PCV (n = 188)	Control (n = 183)	*p*	OR (95% CI)	*p*	OR (95% CI)	*p*	OR (95% CI)
−625G>A	rs11200638	109/63/23	70/84/33	38/90/55	1.16 × 10^−5^	3.21 (1.88–5.50)	0.005	2.01 (1.23–3.28)	0.106	1.60 (0.90–2.85)
−487T>C	rs2672598	162/31/2	109/67/10	97/68/18	1.31 × 10^−4^	10.53 (2.41–46.04)	0.106	1.92 (0.86–4.28)	0.015	5.48 (1.19–25.37)
c.34delCinsTCCT	–	0/2/193	0/6/182	0/16/167	0.001	0.13 (0.03–0.57)	0.029	0.34 (0.13–0.90)	0.220	0.38 (0.08–1.89)
c.59C>T	–	3/42/150	3/31/153	2/56/125	0.130	0.69 (0.43–1.11)	0.004	0.49 (0.30–0.79)	0.190	1.42 (0.84–2.31)
c.102C>T	rs1049331	108/64/23	70/81/34	38/90/55	1.16 × 10^−5^	3.21 (1.88–5.50)	0.009	1.91 (1.17–3.11)	0.072	1.68 (0.95–2.99)
c.108G>T	rs2293870	108/64/23	70/81/34	38/90/55	1.16 × 10^−5^	3.21 (1.88–5.50)	0.009	1.91 (1.17–3.11)	0.072	1.68 (0.95–2.99)

The genotypic distribution was presented as homozygous/heterozygous/wild type. The odds ratios were assessed under dominant model.

**Table 3:** Haplotype-based association analysis of *HTRA1* polymorphisms.

Haplotypes	AMD vs Control			PCV vs Control			AMD vs PCV		
	Ratio	*p*	OR (95% C.I.)	Ratio	*p*	OR (95% C.I.)	Ratio	*p*	OR (95% C.I.)
ACCTT	0.718:0.451	<0.0001	3.10 (2.29–4.19)	0.577:0.450	0.0007	1.66 (1.24–2.22)	0.718:0.578	<0.0001	1.87 (1.38–2.52)
GTCCG	0.090:0.284	<0.0001	0.25 (0.16–0.38)	0.219:0.283	0.0128	0.71 (0.51–1.00)	0.089:0.217	<0.0001	0.35 (0.23–0.54)
GCTCG	0.121:0.164	0.1677	0.70 (0.46–1.06)	0.100:0.163	0.0500	0.57 (0.37–0.89)	0.120:0.100	1.0000	1.22 (0.78–1.92)
GCCCG	0.069:0.096	0.3826	0.70 (0.42–1.19)	0.081:0.098	0.1232	0.65 (0.38–1.11)	0.070:0.066	1.0000	1.04 (0.59–1.84)

The haplotypes were defined by rs11200638, rs2672598, c.59C>T, rs1049331 and rs2293870. The *p*-value was corrected by permutation test (n = 10,000).
