# Supplementary material for: HTRA1 promoter variant differentiates polypoidal choroidal vasculopathy from exudative age-related macular degeneration
Source: Sci Rep. 2016 Jun 24;6:28639. doi: 10.1038/srep28639 (PMC4919652; doi:10.1038/srep28639)
Supplement: Supplementary Information [file srep28639-s1.doc]

***HTRA1* promoter variant differentiates polypoidal choroidal vasculopathy from exudative age-related macular degeneration**

Tsz Kin Ng,^1,*^ Xiao Ying Liang,^1,*^ Timothy Y.Y. Lai,^1^ Li Ma,^1^ Pancy O.S. Tam,^1^ Jian Xiong Wang,^1^ Li Jia Chen,^1^ Haoyu Chen,^2^ Chi Pui Pang.^1^

^1^ Department of Ophthalmology and Visual Sciences, The Chinese University of Hong Kong, Hong Kong.

^2^ The Joint Shantou International Eye Center of Shantou University and The Chinese University of Hong Kong, Shantou, China.

* These two authors contributed equally to this work.

**Correspondence and reprint:**

**Prof. C. P. Pang**

Department of Ophthalmology and Visual Sciences, The Chinese University of Hong Kong, 4/F, Hong Kong Eye Hospital, 147K Argyle Street, Kowloon, Hong Kong.

Phone: +852-39435801; FAX: +852-27159490; E-mail: cppang@cuhk.edu.hk

**Supplementary table 1: The odds ratio analysis of *HTRA1* rs11200638 and rs2672598 joint genotypes**

|  | **Exudative AMD** | | |
| --- | --- | --- | --- |
| **rs11200638** | **rs267598** | | |
|  | **TT** | **TC** | **CC** |
| **GG** | 1.00 (reference) | 4.00 (0.44 - 36.08) | 18.00 (2.00 - 161.83) |
| **GA** | − | 5.27 (0.64 - 43.23) | 12.86 (1.64 - 100.86) |
| **AA** | − | − | 43.11 (5.56 - 334.49) |
|  | | | |
|  | **PCV** | | |
| **rs11200638** | **rs267598** | | |
|  | **TT** | **TC** | **CC** |
| **GG** | 1.00 (reference) | 1.19 (0.43 - 3.26) | 1.60 (0.47 - 5.46) |
| **GA** | − | 2.54 (1.03 - 6.23) | 1.27 (0.51 - 3.17) |
| **AA** | − | − | 3.68 (1.51 - 8.99) |

The odds ratios were not shown since the count of the respective joint genotypes is 0.

The GG-TT joint genotype was used as a reference.

**Supplementary Table 2: The age and gender distribution of the study subjects in *HTRA1* sequencing study**

|  | **Exudative AMD**  **n = 195** | **PCV**  **n = 188** | **Controls**  **n = 183** |
| --- | --- | --- | --- |
| **Males, n (%)** | 88 (54.0%) | 134 (72.4%) | 91 (49.7%) |
| **Females, n (%)** | 75 (46.0%) | 51 (27.6%) | 92 (50.3%) |
| **Age range (Years)*** | 60-94 | 43-87 | 60-99 |
| **Mean age ± SD (Years)** | 75.5 ± 7.5 | 68.2 ± 9.0 | 73.3 ± 6.5 |

* Age of presentation; SD: standard deviation
